# Anatomic Evaluation of the Internal Mammary Vessels Using Multidetector CT Angiography Images in 100 Asian Patients

**Published:** 2014-02-06

**Authors:** Hiroki Tashima, Masakazu Fujikawa, Ken Izumi, Ken Matsuda, Koichi Tomita, Ko Hosokawa

**Affiliations:** ^a^Department of Plastic and Reconstructive Surgery, Osaka General Medical Center, Osaka, Japan; ^b^Department of Plastic and Reconstructive Surgery, Graduate School of Medicine, Osaka University, Osaka, Japan

**Keywords:** anatomical variations, internal mammary vessels, breast reconstruction, multidetector computed tomography, Asian patients

Dear Sir,

Internal mammary vessels (IMVs) are used extensively as recipient vessels for microsurgical breast reconstruction.^1^^-^3 Advantages of IMVs include sufficient arterial flow and easy flap positioning when using flaps with a short pedicle,^1^^,^^4^ although an inadequate diameter of the internal mammary vein may compel surgeons to select an alternative recipient site, such as thoracodorsal vessels. Evaluation of anatomical variations of IMVs is thus important to ensure safe free-tissue transfer. Recent advances in multidetector computed tomography (MDCT) made possible minimally invasive visualization of vessels with very small diameters. We evaluated normal anatomical features and variations of IMVs in 100 Asian patients who underwent MDCT angiography of the chest for various reasons.

All study procedures were approved by the institutional review board. A total of 100 female patients (mean age, 56.3 years; range, 26–77 years) who underwent iopamidol-enhanced MDCT angiography of the chest from April 2012 through October 2012 for various reasons (patients with cancer in various organs, 88; other patients, 12) were analyzed retrospectively. Axial images were scanned by a 64-MDCT scanner (Aquilion 64, Toshiba Medical Systems) and transferred to an office workstation (Ziostation, AMIN Co Ltd). Multiplanar-reformatted, 3-dimensional volume rendering images were used for evaluation. Anatomical features and the internal diameter of IMVs on right and left sides in the second to fifth intercostal space (ICS) just beneath the upper costal edge were evaluated by 2 physicians. A Student *t* test was used to assess differences in diameter between right and left IMVs, and the chi-squared test for the incidence of venous duplication. *P* < 0.05 was considered statistically significant.

The most common variation according to the modified Schwabegger classification^5^^,^^6^ was type I (left: 65%, right: 54%), followed by type II (left: 40%, right: 20%); types III, IV, and V were relatively rare (left: 1%, 3%, and 2%; right: 1%, 4%, and 10%, respectively). The most common feature (95%) consisted of the internal mammary vein running medial and parallel to the artery. Consistent with previous reports,^2^^,^^3^^,^^7^ the diameter of the internal mammary vein was significantly smaller on the left side than on the right side (*P* < .001) (Fig 1a), whereas no significant difference in diameter was observed between left and right internal mammary arteries (Fig 1b). In the third ICS, 62% and 72% of left- and right-side veins, respectively, had a diameter greater than 2.5 mm. In the fourth ICS, right-side veins measured greater than 2 mm in diameter in most patients, whereas left-side veins measured greater than 2 mm only in 26% of patients. The incidence of venous duplication in the second, third, fourth, and fifth ICS was 2%, 35%, 50%, and 56% on the left side, respectively, and 10%, 35%, 62%, and 75% on the right side, respectively. No significant difference was observed in the incidence of venous duplication between left and right sides.

Resection of multiple rib segments for intraoperative anatomic considerations may increase the rate of postoperative contour deformities that are difficult to correct.^8^ Preoperative detection of stable recipient vessels using enhanced-MDCT angiography would help eliminate risks and ensure safety. However, when such information is not available, our results suggest that the most consistent ICS for anastomosis in Asian patients would be the third ICS, which offers adequate venous diameters, given that IMVs in the second ICS are often difficult to access under the mastectomy flap.^9^

## Figures and Tables

**Figure 1:**
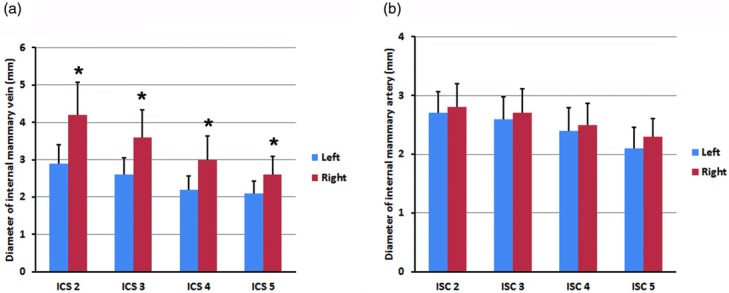
Diameters of the internal mammary vein (*a*) and artery (*b*) were measured in the second to fifth intercostal space. Data are expressed as mean ± SD. *P < 0.001, Student *t* test.
